# Research Note: Transcriptome analysis reveals differentially expressed genes regulated muscle development in Pekin ducks during dietary threonine deficiency

**DOI:** 10.1016/j.psj.2023.103168

**Published:** 2023-10-11

**Authors:** Wenqian Jia, Lei Wu, Zhong Zhuang, Minghong Xu, Yijia Lu, Zhixiu Wang, Hao Bai, Guohong Chen, Guobin Chang, Yong Jiang

**Affiliations:** ⁎ Key Laboratory of Animal Genetics and Breeding and Molecular Design of Jiangsu Province, College of Animal Science and Technology, Yangzhou University, Yangzhou 225009, China; † Joint International Research Laboratory of Agriculture and Agri-Product Safety, the Ministry of Education of China, Institutes of Agricultural Science and Technology Development, Yangzhou University, Yangzhou 225009, China

**Keywords:** threonine, breast muscle, transcriptome, duck

## Abstract

To investigate the underlying molecular mechanism of threonine (**Thr**) regulation on the development of breast muscle in Pekin ducks, 240 male Pekin ducks at 1 d of age were fed a Thr deficiency diet (**Thr-D**), Thr sufficiency diet (**Thr-S**), or Thr excess diet (**Thr-E**) for 21 d. The results showed that Thr-D reduced body weight (**BW**), average weight gain (**ADG**), and average feed intake (**ADFI**), and increased the feed/gain (**F/G**) in Pekin ducks (*P* < 0.05), and Thr-E did not affect BW, ADG, ADFI, or F/G (*P* > 0.05), compared with Thr-S. The diameter and cross-sectional area of the breast muscle fibers in the Thr-S group were larger than those in the Thr-D group (*P* < 0.05). RNA sequencing revealed 1,300 differential expressed genes (**DEGs**) between the Thr-D and Thr-S groups, of which 625 were upregulated and 675 were downregulated by Thr-D. KEGG analysis showed that the upregulated genes were enriched in mTOR, FoxO, Wnt, fat digestion and absorption, and other signaling pathways. The downregulated genes were enriched in the MAPK signaling, glycolysis/gluconeogenesis, adipocytokine signaling, and biosynthesis of unsaturated fatty acids signaling pathways. The genes of Wnt family member 3a (***Wnt3a***), myogenin, myozenin 2, and insulin like growth factor 2 mRNA binding protein were upregulated, and platelet derived growth factor subunit B, PDGF receptor beta and *Wnt4* were downregulated by Thr deficiency, which involving in muscle development. Our findings indicated that Thr increased breast fiber size, perhaps because Thr affected the proliferation and differentiation of satellite cells in breast muscle of ducks after hatch. Our results provide novel insights into new understanding of the molecular mechanisms underlying breast muscle development in ducks subjected to dietary Thr.

## INTRODUCTION

Threonine (**Thr**) is the third limited amino acid in poultry and plays a major role in many metabolic processes, including increasing feed intake, improving growth, maintaining energy homeostasis, enhancing immunity, and improving fat metabolism (Jiang et al., 2016, 2019; Zhang et al., 2016). Previous study showed dietary Thr deficiency or excess reduces protein synthesis of skeletal muscles in young pigs (Wang et al., 2007), which indicated Thr might affect skeletal muscle development. Zhang et al. (2016) found that dietary Thr supplementation increase muscle weight and percentage in ducks. A previous study from our lab also showed that dietary Thr supplementation increases the breast muscle percentage (Jiang et al., 2019).

The molecular mechanisms of skeletal muscle development involve the interaction of multiple signaling pathways. Previous studies have shown that Notch, Wnt, mTOR, MAPK, IGF1, and FOXO signaling pathways regulate muscle satellite cell (**SC**) fate selection, muscle fiber type transformation, and formation of skeletal muscle after hatching (Foletta et al., 2011; Dukes et al., 2015; Deng et al., 2019). Previous study showed leucine promote protein synthesis in skeletal muscle of rats by increasing phosphorylation level of S6K1 in mTOR pathway (Averous et al., 2012), and promote porcine myoblast differentiation through Akt/FoxO1 signaling pathway (Zhang et al., 2018). Lysine promoted skeletal muscle growth of porcine though activating muscle SCs with mTOR signaling pathway (Jin et al., 2020). Methionine improved the breast muscle yield of broiler probably through mTOR, FOXO and IGF1 pathway (Wen et al., 2014a,b). However, the underlying molecular changes in the breast muscle caused by Thr remain unclear.

Therefore, the present study was conducted to demonstrate the effects of dietary Thr levels on growth performance, fiber size, and gene expression profiles in the breast muscle of ducks and to explore potential pathways and key genes whereby Thr increases breast muscle development in Pekin ducks.

## MATERIALS AND METHODS

### Ethics Statement

The study was approved by the Animal Management Committee (in charge of animal welfare issues) of the Institute of Animal Science, Chinese Academy of Agricultural Sciences (IAS 2020-114) and was performed in accordance with the guidelines. Ethical approval for animal survival was provided by the Animal Ethics Committee of IAS-CAAS.

### Experimental Animals, Treatments, and Diets

A total of 240 one-day-old male Pekin ducks with similar body weights were randomly divided into 3 groups with 8 replicate cages of 10 ducks per cage (the average body weight of per cage was about 56.8±0.47 g at 1 d of age). The ducks were fed corn-wheat-peanut meal basal diet or basal diet supplemented with 0.25 and 0.50% crystalline Thr from 1 to 21 d of age. Birds were housed in raised wire-floor cages under 24 h of continuous light during the experimental period. The cage was equipped with nipple and tubular feeders. Room temperature was maintained at 30°C from 1 to 3 d of age, and then gradually decreased to 25°C until 21 d of age.

The basal diet contained 24.8% corn, 20.0% peanut meal, 48.5% wheat, 1.13% limestone, and 1.85% dicalcium phosphate. We also balanced the methionine, tryptophan, lysine, valine, and isoleucine in base diet. The dietary protein level is about 20% in basal diet, and dietary Thr concentrations were 0.41, 0.66, and 0.91% for the Thr deficiency treatment (**Thr-D**), Thr suite treatment (**Thr-S**), and Thr excess treatment (**Thr-E**), respectively.

### Sample Collection

At 21 d of age, experimental ducks fasted for 12 h, then were recorded the body weight of each cage, residual feed, and calculated the average daily gain (**ADG**), average daily feed intake (**ADFI**), and feed/gain (**F/G**) for 1 to 21 d. Three ducks from each cage were selected according to their average body weight, and were euthanized via CO_2_ inhalation. Breast muscle samples (from similar region) were quickly separated, frozen in liquid nitrogen, and stored at −80°C for transcriptomics. Another part of the breast muscle was separated and fixed in 4% formalin for histological examination.

### Histological Evaluation

Breast muscles were fixed in 4% neutralized formalin, embedded in paraffin blocks, sectioned, and the muscle fiber diameter and area were measured using a biological microscope to estimate the size of the muscle fiber. Muscle fiber morphological trait measurements of each muscle sample were determined using the method described by Weng et al. (2021).

### Transcriptome Sequencing

Total RNA from frozen breast muscle of Pekin ducks was isolated using the RNAiso Plus kit (Takara, Dalian, China) following the recommended protocol. Each qualified sample was prepared with 3 μg RNA for library construction. Pair-end sequencing of the samples was performed at Berry Genomics (Beijing, China) using an Illumina X-TEN (Illumina, California). To ensure the quality of the information analysis, FASTQC software was used to control the quality of the original data (raw reads). Reads that only contained sequencing adapter sequences and those that contained N ratios were excluded. Reads with more than 0.1% and low-quality reads were removed, thus obtaining to high-quality sequence data (clean reads). Clean data were obtained after removing connectors and low-quality reads from the original data. The clean data were compared with the duck reference genome using Bowtie software to obtain the position and characteristic information of the sequence in the duck reference genome.

### Analysis of Differentially Expressed Genes and Pathway Enrich

Differentially expressed genes (**DEGs**) in breast muscle between the Thr-D and Thr-S groups were analyzed using R software with the EdgeR package. |log^FC^| > 1 and FDR < 0.05, as the thresholds to screen for DEGs. Normalized gene expression values are presented as counts per million (**CPM**). A heat map was used to construct a cluster among the samples according to the CPM of all genes. DEGs were converted to their FASTA protein sequences, and gene enrichment was performed using the FASTA Protein Sequences by Kyoto Encyclopedia of Genes and Genomes (**KEGG**) significant enrichment analysis in Kobas 3.0 (http://kobas.cbi.pku.edu.cn/index.php).

### Statistical Analyses

Data from the experiment were subjected to 1-way ANOVA using the general linear model procedure of SAS software (SAS Institute Inc., Cary, NC; 2003). Differences among means were tested using Duncan's method. The level of statistical significance was set at *P*< 0.05.

## RESULTS AND DISSCUSION

**Figure 1:**
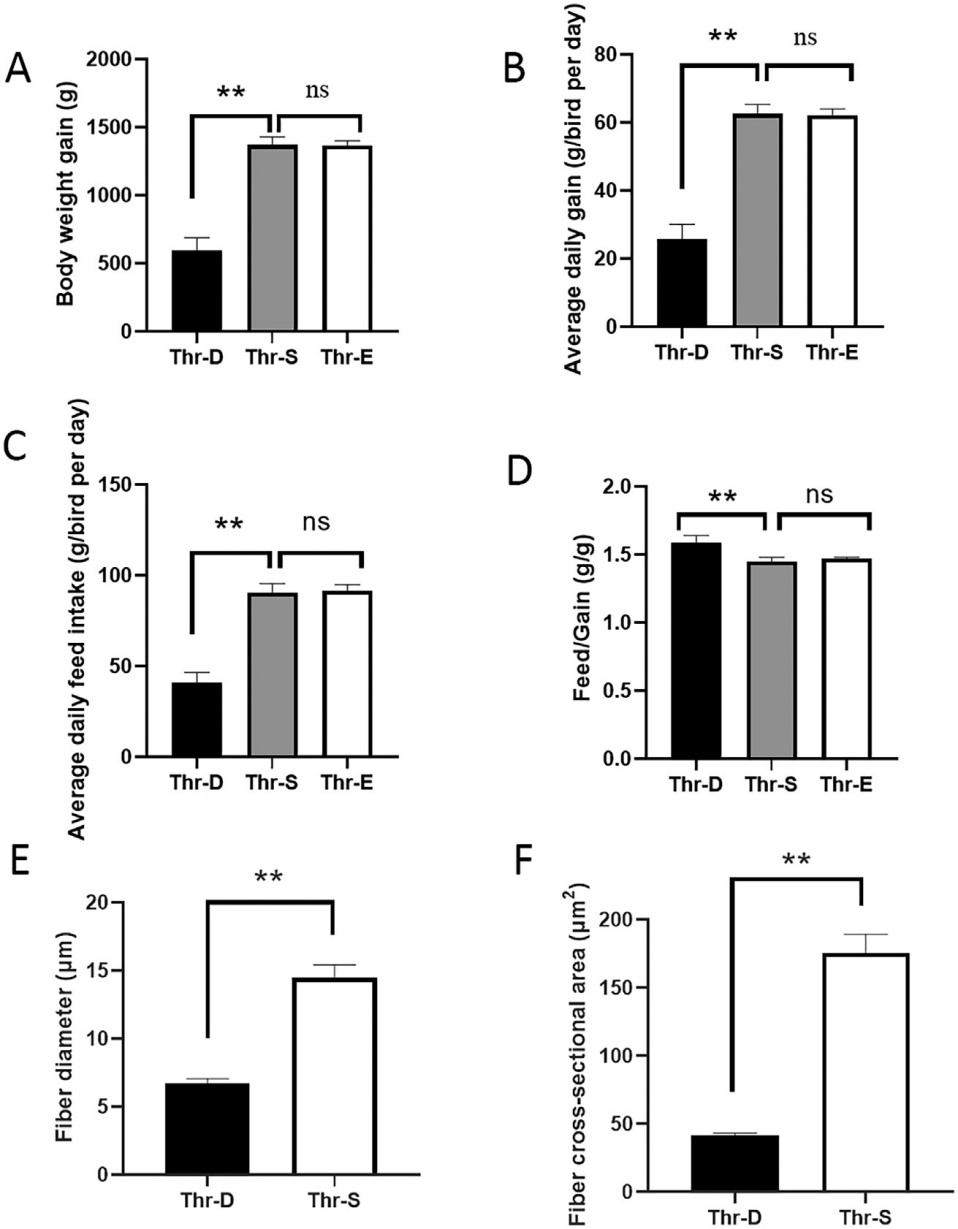
Growth performance and breast muscle phenotype in 21-day ducks. (A) Body weight. (B) The average daily gain. (C) The average daily feed intake. (D) The feed/gain. (E) Muscle fiber diameter. (F) Cross-sectional area. * *P* < 0.05; ** *P* < 0.001; ns, no significance. Thr-D, threonine deficiency; Thr-S, threonine sufficiency; Thr-E, threonine excess.

Thr-D decreased body weight (**BW**), ADG, and ADFI (*P* < 0.05) and increased the F/G (*P* < 0.05), and Thr-E had no influence on growth performance in present study (Figure 1A–D). The BW in Thr-S group was 1.79 times higher than that in Thr-D group (Figure 1A). These results were agreed with the previous studies (Zhang et al., 2016). A previous study showed that dietary supplementation with appropriate Thr can improve breast muscle percentage of ducks (Jiang et al., 2019). The results from present study showed Thr-S increase the diameter and cross-sectional area of the breast muscle fibers than that in Thr-D group (*P* < 0.05, Figure 1E and F). Zhang et al. (2016) also found supplementation of Thr increase breast muscle yield. However, the underlying molecular changes in the breast muscle caused by Thr remain unclear.

**Figure 2:**
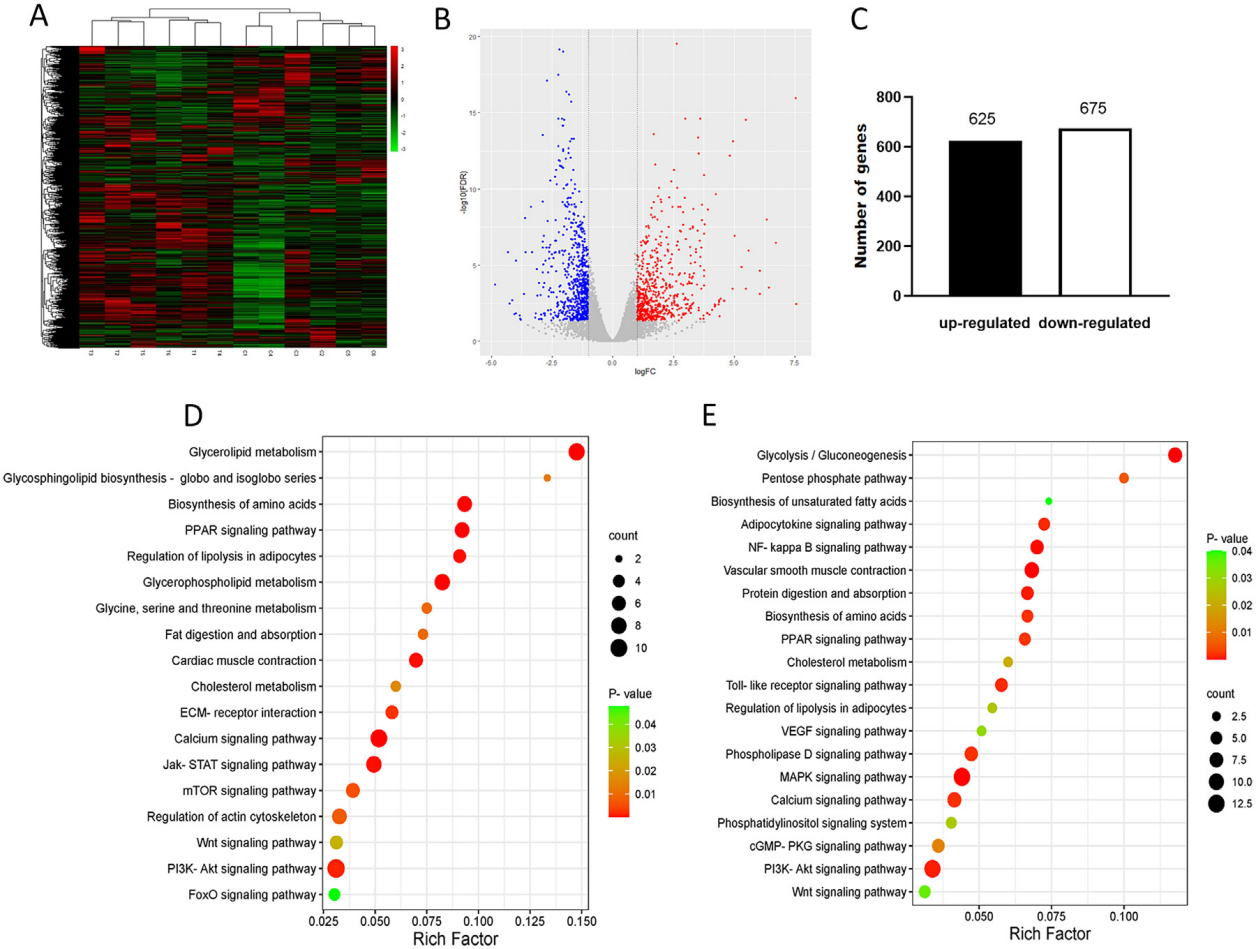
Differential gene and gene function enrichment analysis. The (A) Heatmap, T1 to T6 are the 6 the samples in the Thr-D group. (B) Volcano plot. (C) Number of differentially expressed genes. (D) Bubble plot for KEGG-enriched pathway of upregulated gene enrichment; (E) Bubble plot for KEGG-enriched pathway of downregulated gene enrichment.

Therefore, we screened gene expressed profiles in breast muscle using RNA sequencing techniques to explore the potential pathways and key genes by compared the DEGs between Thr-S and Thr-D groups (6 vs. 6). The samples from the Thr-D and Thr-S groups were clustered into 2 categories by Heat Cluster Analysis (Figure 2A). DEGs were identified using a *t* test in R software with EdgeR package (|FDR| < 0.05, and |log^FC^| > 1). There were 1,300 DEGs between the Thr-D group and the Thr-S group, of which 625 genes were upregulated and 675 genes were downregulated in the Thr-D group compared to the Thr-S group (Figure 2B and C). The results of KEGG enrichment showed that Thr upregulated genes were also enriched in mTOR, FoxO, Wnt, fat digestion, and absorption, etc. (Figure 2D). The downregulated genes were enriched in the MAPK signaling pathway, Wnt, glycolysis/gluconeogenesis, adipocytokine signaling pathway, etc. (Figure 2E). These pathways deserve further study to help better understand the molecular mechanism by which Thr regulates muscle development in Pekin ducks, and to further refine it.

After hatching, skeletal muscle development and muscle regeneration in animals mainly rely on SCs in skeletal muscle. The classical Wnt signaling pathway mainly regulates the proliferation and differentiation of skeletal muscle SCs (Kamizaki et al., 2021). Previous studies have shown that Wnt family member 3a (***Wnt3a***) regulates the function of myogenic differentiation antigen (***MYOD***) and myogenin (***MYOG***) (Tajbakhsh et al., 1998), which are highly expressed genes in fast and slow muscle fibers, respectively. Overexpression of *Wnt3a* promotes the expression of myosin heavy chain class I (***MyHC-I***) in slow muscle fibers of skeletal muscle, and promotes the differentiation of cells to slow muscle fibers (Kuroda et al., 2013). Wnt family member 4 (***Wnt4***) can activate *MYOD* expression, and promotes the formation of fast muscle fiber in the early stage of muscle fiber formation in chicks (Kamizaki et al., 2021). Myozenin 2 (**MYOZ2**) belongs to the MYOZs family and mainly expressed in slow muscle and cardiac muscle. Previous studies have shown that knockout of the mice *MYOZ2* resulted in significant enhancement of calmodulin phosphatase activity, which in turn stimulated the CaN-NFAT signaling pathway and initiated the mechanism of generating slow muscle fibers, resulting in an increase in the proportion of slow muscle fibers (Frey et al., 2004). In Pekin ducks, *MYOZ2* expression in breast muscle showed differences between Clostridium butyricum-treated and control groups (Liu et al., 2018). Thr deficiency upregulated *Wnt3a, MYOG*, and *MYOZ2* expression and downregulated *Wnt4* expression, suggesting that Thr might regulates skeletal muscle development of ducks by Wnt singing pathway.

A previous study found that insulin like growth factor 2 mRNA-binding protein (***IGF2BP1***) is a key gene regulating the body size of Pekin ducks (Zhou et al., 2018). Overexpression of *IGF2BP1* reduced the mRNA levels of *MYOD, MYOG*, and myogenic factor 5 (***Myf5***) in the leg muscle of chickens and also reduced the protein expression of MYOD, indicating that high expression of *IGF2BP1* in chicken skeletal muscle may inhibit muscle proliferation and differentiation (Feng et al., 2021). In addition, Smith et al. (1999) found that platelet derived growth factor subunit A (**PDGF-A**) and platelet derived growth factor subunit B (**PDGF-B**), members PDGF, promote the proliferation of SC via binding with PDGF receptor beta (**PDGFRB**). In this study, The Thr-deficient diet downregulated the gene expression of *PDGF-B* and *PDGFRB* and upregulated *IGF2BP1* expression. Therefore, we speculated that Thr supplementation into diets affect the skeletal muscle development of Pekin ducks after hatching possibly by inducing SC differentiation.

In conclusion, dietary Thr improved the growth performance and breast muscle fiber size of Pekin ducks, and affected gene expressions of *Wnt3a, Wnt4, MYOG, MYOZ*2, *PDGF-B, PDGFRB*, and *IGF2BP1*, which regulating proliferation and differentiation of SC in breast muscle. These findings provide novel insights into understanding of the molecular mechanism of the regulation of Thr on breast development in Pekin ducks after hatching.
